# Juvenile Hemochromatosis Connecting Cardiac Arrest and Hypogonadotropic Hypogonadism in a Young Woman

**DOI:** 10.1210/jcemcr/luae196

**Published:** 2024-10-24

**Authors:** Kevin Alexander Zinecker, Aleksandra Sliwinska, Leah M Wilson, Roula Zahr

**Affiliations:** Department of Internal Medicine, Oregon Health Sciences University Hillsboro Medical Center, Hillsboro, OR 97123, USA; Department of Endocrinology Diabetes and Clinical Nutrition, Oregon Health Sciences University, Portland, OR 97239, USA; Department of Endocrinology Diabetes and Clinical Nutrition, Oregon Health Sciences University, Portland, OR 97239, USA; Department of Endocrinology Diabetes and Clinical Nutrition, Oregon Health Sciences University, Portland, OR 97239, USA

**Keywords:** hereditary hemochromatosis, juvenile hemochromatosis, *HJV* gene, endocrinopathy, hypogonadotropic hypogonadism, cardiomyopathy

## Abstract

Juvenile hemochromatosis (JH) is a subtype of hereditary hemochromatosis, a genetic disorder characterized by excessive iron absorption and deposition in various organs, leading to cardiomyopathy, cirrhosis, and diabetes. Endocrine dysfunction is a common manifestation and may appear years before end-organ damage. This report describes a rare case of JH, emphasizing the consequences of delayed diagnosis. A 28-year-old woman with a history of hypogonadotropic hypogonadism presented with cardiac arrest complicated by acute renal failure and cerebral vascular accidents. She initially exhibited signs of severe cardiomyopathy and multiorgan failure, which led to workup for an underlying cause. Laboratory values showed significantly elevated ferritin and transferrin saturation. Subsequent genetic screening revealed *HJV* gene pathogenic variants consistent with juvenile hemochromatosis. Treatment involved aggressive iron chelation therapy and outpatient referral for cardiac transplant. This case calls for heightened awareness and early screening of JH, particularly among patients with unexplained endocrine dysfunction. Early diagnosis and treatment are paramount in preventing irreversible organ damage and improving patient outcomes.

## Introduction

Hereditary hemochromatosis (HH) is primarily an autosomal recessive genetic condition, which causes excess iron deposition in vital organs and tissues, often manifesting in later stages as cardiomyopathy, cirrhosis, diabetes, and endocrine dysfunction. Expression of several pathogenic variants leads to subtypes of this disease, dictating age of onset, clinical manifestations, and disease severity. The incidence in non-Hispanic White people is as high as 1 in 200, although the phenotypic penetrance is low [1]. Type 1 HH is the most prevalent subtype and is caused by pathogenic variants in the *HFE* gene (C282Y or H63D pathogenic variants); it typically presents in the fourth or fifth decade of life. Two less common subtypes, termed “juvenile hemochromatosis” (JH), are caused by pathogenic variants in the hemojuvelin gene (*HJV* type 2a HH) or hepcidin gene (*HAMP* type 2b HH), with onset frequently in the second to third decade of life. Pathogenic variants in the *TfR2* gene (type 3 HH) and ferroprotein-related genes (type 4 HH) are rare [2]. Endocrinopathies in hemochromatosis are common and can precede liver, heart, or other end-organ damage [2]. Here we report a case of a young woman with hypogonadotropic hypogonadism and delayed diagnosis of JH resulting in multiorgan failure.

## Case Presentation

A 28-year-old female with a past medical history of amenorrhea due to hypogonadotropic hypogonadism presented to the hospital in critical condition after a witnessed out-of-hospital cardiac arrest. With resuscitation efforts, a return of spontaneous circulation was obtained in the field. In the emergency department she was found to have acute hypoxic respiratory failure requiring intubation, shock requiring vasopressors, and complete heart block requiring transvenous pacing. Echocardiogram revealed severe biventricular heart failure and left ventricular thrombus, ultimately leading to implantable cardioverter defibrillator placement. The hospital course was complicated by acute renal failure requiring continuous renal replacement therapy and an ischemic right middle cerebral artery cerebrovascular accident with hemorrhagic conversion, for which she underwent thrombectomy.

Family members reported 4 months of progressively worsening exertional dyspnea and 2 years of lower extremity swelling prior to the event. Her substance use history included marijuana and nicotine use but no heavy alcohol or intravenous drug use. As a child, she participated in tennis and cheerleading. She had no physical limitations compared to her peers until recent months. There was no family history of cardiac pathology.

## Diagnostic Assessment

Transthoracic echocardiogram demonstrated severely reduced left and right ventricular systolic function with left ventricular ejection fraction <20% and increased right ventricle wall thickness. Unexplained heart failure prompted evaluation with left and right heart catheterization, which was negative for coronary artery disease. Up-trending liver enzymes were attributed to decompensated heart failure and other causes were initially excluded. Iron studies were performed as part of the anemia workup, and elevated ferritin was felt to be secondary to acute illness at that time [Table 1]. Cardiac positron emission tomography-computed tomography was negative for sarcoidosis, inflammatory myocarditis, or malignancy. Initial hemochromatosis mutation analysis showed neither of the 2 alleles of the *HFE* gene contained the C282Y or H63D pathogenic variants. Ultimately, the decision was made to pursue endomyocardial biopsy. Surgical pathology revealed increased iron deposition in the cardiomyocytes consistent with cardiac hemochromatosis. A follow-up hereditary hemochromatosis genetic panel analyzed *FTH1*, *FTL*, *HAMP*, *HFE*, *HJV*, *SLC40A1*, and *TFR2* genes. Results found homozygosity in the *HJV* gene for a sequence variant defined as c.959G > T, which is predicted to result in amino acid substitution p.Gly320Val (OMIM 608374), confirming the diagnosis of JH.

**Table 1.** Laboratory values on admission

Laboratory test	International system (and conventional) units	Normal range
Hemoglobin	6.7 mmol/L (10.8 g/dL)	7.4-9.9 mmol/L (12-16 g/dL)
Lactic acid	8 mmol/L (72 mg/dL)	≤2.0 mmol/L (≤18 mg/dL)
pH	7.1	7.35-7.45
Bicarbonate	14.5 mmol/L (88.5 mg/dL)	22-26 mmol/L (134-159 mg/dL)
Brain natriuretic peptide	553 pmol/L (4674 pg/mL)	0-11.8 pmol/L (0-100 pg/mL)
High sensitivity troponin	Initial: 385 mcg/L (385 ng/L) 2-hour repeat: 14 218 mcg/L (14 218 ng/L)	≤75 mcg/L (≤75 ng/L)
Ferritin	35 nmol/L (15 710 ng/mL)	0.11-0.26 nmol/L (50-114 ng/mL)
Transferrin saturation	116%	20-50%
Iron level	43.3 µmol/L (2418 ng/mL)	14-32 µmol/L (782-1787 ng/mL)

Further history revealed thelarche and adrenarche at age 11 and menarche at age 12 with regular menses until age 14, when she developed amenorrhea and hot flashes and was diagnosed with hypogonadotropic hypogonadism. At that time, mildly elevated liver enzymes were not further investigated [Table 2] and pituitary magnetic resonance imaging was normal without signs of iron deposition. Estradiol levels were not checked. The patient had no history of anosmia or other hormonal deficiencies and no family history of hypogonadism. Low FSH and LH were attributed to functional hypogonadotropic hypogonadism in the setting of potentially vigorous exercise, although body mass index was 20 kg/m^2^. Subsequently, the patient was started on combined oral contraceptive pills without interruption in use.

**Table 2.** Laboratory values at age 14

Laboratory test	International system (and conventional) units	Normal range
Alanine aminotransferase	2.47 µkat/L (149 U/L)	0-0.58 µkat/L (14-72 U/L)
Aspartate aminotransferase	1.03 µkat/L (62 U/L)	0-0.58 µkat/L (9-40 U/L)
Prolactin	5.1 µg/L (5.1 ng/mL)	0-40 µg/L (2.9-29.2 ng/mL)
TSH	1.3 mIU/L (1.3 µIU/mL)	0.5-4.70 mIU/L (0.5-4.70 µIU/mL)
Free T4	14.2 pmol/L (1.1 ng/dL)	10-23 pmol/L (0.8-2.2 ng/dL)
FSH	2.7 IU/L (2.7 mIU/mL)	2.5-116.3 IU/L (2.5-116.3 mIU/mL)
LH 2.6 IU/L	2.6 IU/L (2.6 mIU/mL)	<0.5-54 IU/L (<0.5-54 mIU/mL)
Free testosterone	2.1 pmol/L (0.6 pg/mL)	3.5-31.2 pmol/L (1.2-7.5 pg/mL)
Total testosterone	0.3 nmol/L (9 ng/dL)	0.17-1.11 nmol/L (9-49 ng/dL)
SHBG	125 nmol/L (1187 µg/L)	11-120 nmol/L (1045-11400 µg/L)

On hospital day 38, endocrinology was consulted for evaluation of endocrine dysfunction related to JH and obtained additional laboratory values [Table 3]. An examination was notable for tanned skin. Blood sugars ranged from 80 to 325 mg/dL (4.44-18.0 mmol/L) (65-95 mg/dL, 3.6-5.3 mmol/L) throughout hospitalization and provided a new diagnosis of prediabetes. Thyroid and adrenal axes as well as repeat prolactin levels were normal. In retrospect, the patient's long-standing history of hypogonadal hypogonadism was rather attributed to JH, which commonly causes central hypogonadism [3].

**Table 3.** Laboratory values on endocrine evaluation

Laboratory test	International system (and conventional) units	Normal range
Hemoglobin A1c	6.3% (45 mmol/mol)	<5.7% (<39 mmol/mol)
PTH	19.8 pmol/L (187 ng/L)	1.27-9.33 pmol/L (12-88 ng/L)
Calcium	2.12 mmol/L (8.5 mg/dL)	2.12-2.62 mmol/L (8.6-10.2 mg/dL)
Phosphorus	1.23 mmol/L (3.8 mg/dL)	0.97-1.45 mmol/L (2.4-4.7 mg/dL)
25-(OH) vitamin D	49.9 nmol/L (20 ng/mL)	19.9-199.7 nmol/L (30-80 ng/mL)

## Treatment

Cardiac and liver transplant were considered; however, case reports suggested that medical management could reverse organ damage. Following a hematology consult, the patient was promptly started on treatment with combination chelation therapy with deferoxamine and deferiprone. Therapeutic phlebotomy was not indicated due to concurrent anemia. Ferritin levels decreased to approximately 4000 ng/mL by the time of discharge.

## Outcome and Follow-up

Despite a further decrease of ferritin to less than 1000 ng/mL, repeat transthoracic echocardiogram demonstrated no improvement in ventricular function, which was managed with biventricular pacing and home dobutamine infusions. As a result, cardiology recommended initiating heart transplant and left ventricular assist device evaluation. Hepatology follow-up was arranged to monitor for possible liver involvement of hemochromatosis. An outpatient bone density scan revealed osteoporosis with a T-score of −2.7 in the lumbar spine, −1.8 in the right total hip, and −1.9 in the right femoral neck, and the Z-scores were identical. The patient was started on intravenous zoledronic acid. Repeat pituitary imaging was also planned with endocrinology to review.

## Discussion

JH is caused by pathogenic variants in the hemojuvelin gene (*HJV* type 2a HH) or hepcidin gene (*HAMP* type 2b HH) with onset most frequently before age 30 [3]. Each subtype of hemochromatosis has a variable impact on hepcidin synthesis and function, a protein that closely regulates iron homeostasis, thereby determining the rate and severity of iron accumulation. *HAMP* and *HJV* genes play a larger role in hepcidin function, and pathogenic variants in these genes lead to more rapid iron accumulation and earlier onset of organ dysfunction [1, 4]. Clinical presentation often includes hypogonadotropic hypogonadism, impaired glucose tolerance or diabetes, cardiomyopathy, and cirrhosis, although initial symptoms may be more subtle, with nonspecific fatigue or arthralgias [1]. Cardiac disease is the primary cause of death [3].

Hypogonadism is the most frequent endocrinopathy seen in JH due to iron overload in gonadotrophs [3, 5]. In JH, hypogonadism occurs in 67% to 96% of patients, typically presents with amenorrhea, and often appears years before the onset of cardiac or hepatic manifestations, as witnessed in our case [1, 3]. Amenorrhea is often attributed to the more common functional hypothalamic amenorrhea in young athletic women and cannot be ruled out in our patient. Degree of physical activity, body mass index, and magnetic resonance imaging findings are helpful considerations in making the diagnosis. Gonadotroph dysfunction can recover if HH is treated early in the disease process with phlebotomy [2].

Diabetes is the most common endocrinopathy seen in HH with a prevalence of 13% to 23% [2]. Impaired glucose tolerance occurs more commonly in up to 58% in type 2 HH, compared to 27%, 9.1% in subtypes 1 and 3, respectively [6]. In mouse models, excess iron was shown to cause beta cell damage through oxidant stress and apoptosis, leading to insulin deficiency [7]. Treatment of HH and normalization of ferritin may lead to the reversal of impaired glucose tolerance [8].

A high prevalence of osteoporosis (25-34%) and osteopenia (40-79%) have also been seen in HH, correlating with severity of iron overload regardless of hypogonadism, cirrhosis, or vitamin D deficiency [2, 9]. Studies demonstrated direct toxicity of iron on osteoblast function, which may ameliorate after normalization of ferritin [9, 10]. Our patient was diagnosed with osteoporosis and treated with zoledronic acid, although addressing her underlying condition may effectively improve her low bone mineral density. Less commonly, HH causes dysfunction of the adrenal, thyroid, and parathyroid glands [2].

Screening for HH is not currently recommended for the general population, according to the Hemochromatosis and Iron Overload Screening study [5]. However, it should be considered in those with unexplained heart failure, secondary amenorrhea, liver disease, arthropathy, and fatigue and in patients with atypical presentations of diabetes. Ferritin and transferrin saturation are relatively inexpensive screening tests, and abnormal results may warrant follow-up genetic testing.

This case report aims to bring awareness to the rare diagnosis of JH and highlights the importance of early recognition to prevent end-organ damage, as this condition is life-threatening if left untreated. Endocrine dysfunction is commonly seen in JH, and early intervention may improve hypogonadotropic hypogonadism, impaired glucose tolerance, and bone mineral density.

## Learning Points

- JH is a severe subtype of HH and presents at a young age, leading to earlier organ involvement.
- Diagnosis of JH is often delayed as nonspecific symptoms precede end-organ damage.
- JH may present with hypogonadism years before liver or heart disease.
- Endocrine dysfunction is frequently seen in JH and may be reversible with early treatment.
- Screening is relatively inexpensive and should be considered in patients with a constellation of unexplained symptoms, liver, heart, or endocrine dysfunction.

## Contributors

All authors made individual contributions to authorship. A.S. and R.Z. were involved in the diagnosis and management of the patient. K.A.Z., A.S., L.M.W., and R.Z. were involved in manuscript submission. All authors reviewed and approved the final draft.

## Data Availability

Original data generated and analyzed during this study are included in this published article.
